# Bacterial diversity is strongly associated with historical penguin activity in an Antarctic lake sediment profile

**DOI:** 10.1038/srep17231

**Published:** 2015-11-25

**Authors:** Renbin Zhu, Yu Shi, Dawei Ma, Can Wang, Hua Xu, Haiyan Chu

**Affiliations:** 1 Institute of Polar Environment, School of Earth and Space Sciences, University of Science and Technology of China, Hefei city, Anhui Province 230026, PR China; 2 State Key Laboratory of Soil and Sustainable Agriculture, Institute of Soil Science, Chinese Academy of Sciences, Nanjing, Jiangsu Province 210008, P.R. China

## Abstract

Current penguin activity in Antarctica affects the geochemistry of sediments and their microbial communities; the effects of historical penguin activity are less well understood. Here, bacterial diversity in ornithogenic sediment was investigated using high-throughput pyrosequencing. The relative abundances of dominant phyla were controlled by the amount of historical penguin guano deposition. Significant positive correlations were found between both the bacterial richness and diversity, and the relative penguin number (*p* < 0.01); this indicated that historical penguin activity drove the vertical distribution of the bacterial communities. The lowest relative abundances of individual phyla corresponded to lowest number of penguin population at 1,800–2,300 yr BP during a drier and colder period; the opposite was observed during a moister and warmer climate (1,400–1,800 yr BP). This study shows that changes in the climate over millennia affected penguin populations and the outcomes of these changes affect the sediment bacterial community today.

Antarctic freshwater lakes are relatively simple aquatic ecosystems, with low species richness and diversity, low biomass, short food chains and limited trophic complexity^1^,^2^. However, benthic sediments represent one of the most complex microbial habitats on Earth^3^. Microorganisms in sediments play a significant role in biogeochemical cycles and the remineralisation of organic matter within aquatic ecosystems. For example, bacterial populations are major contributors in the transformation of organic carbon, sulphur, nitrogenous compounds, and metals, and have an important role in ecosystem food webs and nutrient cycling^2^,^4^. A number of freshwater lakes and ponds have formed along the coast of Antarctica due to climate warming and the retreat of the icecap^5^,^6^. This has provided an opportunity to investigate the microbial structure and function in the sediment profiles in these aquatic ecosystems, to understand the role of microorganisms in the Antarctic.

Benthic lake sediments provide a stratified habitat, where the substrates and electron acceptors that are essential for microorganisms are gradually scavenged; as such there are different environmental niches for metabolically diverse microorganisms^7^. Recently, variations in bacterial population structure with sediment depth have been reported in the coastal Pacific^8^, cold seep sediments^9^ and Antarctic sediments^10^,^11^,^12^,^13^. Sediment microbial community structure can be influenced by many environmental factors, such as organic matter, nutrients and salinity^14^,^15^. However, the links between sediment microbial community structure and these environmental factors remain largely unclear^16^,^17^,^18^. Further, information on the depth-related change of sediment microbial community structure is especially limited in Antarctica.

Penguin activity in maritime Antarctica strongly influences the biogeochemistry of soils and sediments, through the deposition of a large amount of penguin guano, which has produced so-called ornithogenic soils and sediments^19^,^20^,^21^,^22^; these soils and sediments are particularly rich in organic carbon, nitrogen, and phosphorus^23^,^24^,^25^,^26^. Microorganisms are crucial for the decomposition and mineralization of organic matter, and carbon and nitrogen biogeochemical cycles in these ornithogenic ecosystems^27^,^28^,^29^,^30^,^31^.

In coastal Antarctica, the bacterial communities in ornithogenic soils have been investigated in the Ross Sea region, Larsman Hills and around Casey Station, and, in general, high soil bacterial diversity has been found^32^,^33^,^34^,^35^. Through analysis of the geochemical elements of the ornithogenic sediment profile on Ardley Island in maritime Antarctica, changes in sediment geochemistry were found to reflect the fluctuations of historical penguin populations^21^,^36^. Molecular biological investigation of the chitinase gene copy numbers suggested that the fluctuation of chitinolytic bacterial numbers correlated with the levels of geochemical elements in the ornithogenic sediment profiles^37^. Bacterial diversity in sediment cores have been analysed using a PCR-Denaturing Gradient Gel Electrophoresis (DGGE) method, revealing a possible link between sediment bacterial composition and the geochemical elements^13^,^35^. However, PCR-DGGE has several limitations, including that only short PCR fragments and band peaks are resolved, which represent several different species that are therefore undifferentiated, because of co-migration in the DGGE gels.

In the previous research on bacterial diversity, as far as we know, the historical penguin population was not constructed to show the correlation between bacterial diversity and penguin activity based on the concentrations of typical elements through the sediment profile. An explicit, quantitative correlation between the stratigraphic variability of geochemical properties and concomitant changes in the structure of the microbial community, or the relative abundance of individual taxa, has remained elusive. It is unclear how historical penguin activity and climate change have influenced shifts in the bacterial community along the ornithogenic sediment depths. Also, research to date has been based on techniques with relatively low taxonomic resolution, and the conclusions reached may have been constrained by the type of analyses; high-taxonomic-resolution techniques can provide detailed phylogenetic-level data.

In this study, one ornithogenic sediment core was collected on Ardley Island in maritime Antarctica during the austral summer of 2011/2012. A high-throughput pyrosequencing method was used to determine the microbial community composition and diversity in the sediment profile. We have tested the following three hypotheses: (i) the vertical distribution patterns of the microbial community composition in the sediment closely correlate with the multivariate biogeochemistry related to penguin activity; (ii) changes in the bacterial community diversity correspond to historical penguin activity in maritime Antarctica; and (iii) the climate change affects not only the penguin population, but also the sediment bacterial community composition and diversity, even over millennia.

## Results

### Sediment geochemical properties

**Figure 1:**
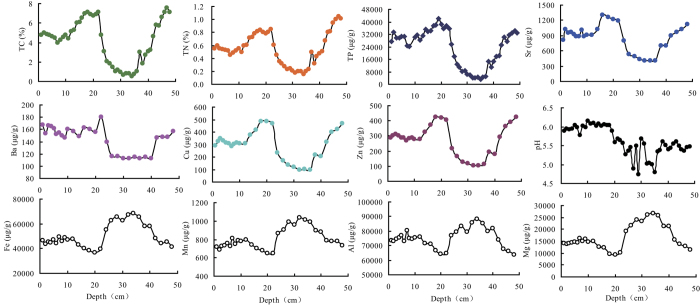
Changes of the geochemical properties with depth in the sediment profile PC of Lake Y2 on Ardley Island in maritime Antarctica.

The concentrations of phosphorus (P), strontium (Sr), barium (Ba), copper (Cu), zinc (Zn), total carbon (TC) and total nitrogen (TN) fluctuated similarly with depth in the ornithogenic sediment profile (PC; Fig. 1). The concentrations of these elements remarkably increased and reached a peak at around 20 cm depth. After the peak, their concentrations dropped quickly and were found at very low levels in the 25–40 cm sediment layers. The element concentrations then increased to a high level in the 40–50 cm layers. The pH of the sediments was acidic (4.8–6.2). The concentrations of iron (Fe), manganese (Mn), aluminium (Al) and magnesium (Mg) all peaked in the 28–40 cm layers, showing an opposite trend to those of the elements P, Sr, Ba, Cu, Zn, TC and TN. Significant positive correlations were found between the concentrations of P, Sr, Ba, Cu, Zn, TC and TN (*p* < 0.01), and significant negative correlations found between those elements and Fe, Mn, Mg and Al (*p* < 0.01; Table S1). The assemblage of these elements, including P, Sr, Ba, Cu and Zn, is an important geochemical characteristic of ornithogenic lake sediments. Overall the vertical distribution patterns of the elements analysed within the sediments were highly consistent with those reported by Sun *et al.* (2000)^21^.

### Bacterial community composition

**Figure 2:**
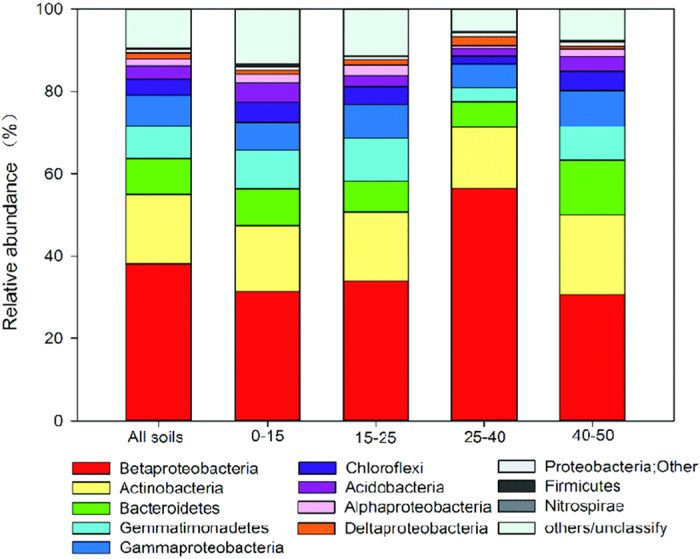
Relative abundance of the dominant bacterial phyla (or class in the case of the phylum Proteobacteria) in the sediments, separated according to sediment depth. The relative abundances are based on the proportional frequencies of the DNA sequences that were classified at the phylum level.

In total, 201,072 high-quality bacterial sequences were obtained with a range of 2,425–12,189 sequences per sample (mean 7,447); we were able to classify 90.4% of these sequences (Table S2). The most dominant bacterial phylum was Proteobacteria (15–78%); within this phylum the class Betaproteobacteria was the most abundant across the sediment profile (mean 38.3%), followed by Gammaproteobacteria (mean 7.1%), Alphaproteobacteria (mean 1.8%) and Deltaproteobacteria (mean 1.4%). The percentages of each class within the phylum Proteobacteria varied greatly with depth in the ornithogenic sediments (Fig. S1). The other dominant phyla in the sediments were: Actinobacteria (mean 16.3%), Bacteroidetes (mean 8.6%), Gemmatimonadetes (mean 7.8%), Acidobacteria (mean 3.3%) and Chloroflexi (mean 4.0%). In total, with the Proteobacteria, these phyla accounted for 87.2% of the bacterial sequences from all of the sediment samples. In addition, Firmicutes (mean 0.2%) and Nitrospirae (mean 0.1%) were present in the sediments, but at relatively low abundances (Fig. 2 and Fig. S2).

**Figure 3:**
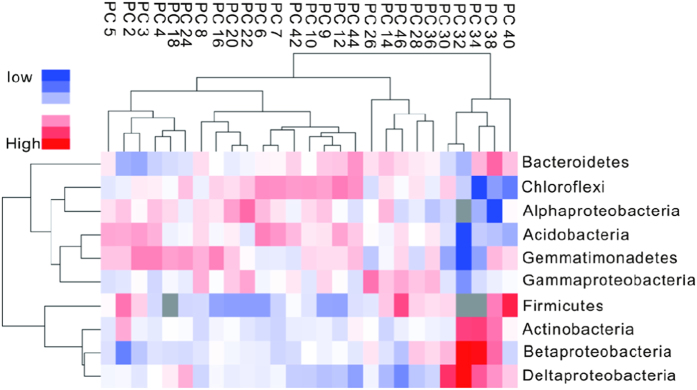
Heatmap diagram of the 10 dominant bacterial phyla among the 27 sediment layers analysed. PC represents the respective layer (depth; cm) of the sediment profile.

Overall the bacterial community structure varied greatly down the sediment profile. According to the sediment depths of the samples, the bacterial community composition could be clustered into two distinctive groups: the first group occurred in the top 1–30 cm and lower 40–50 cm sediment layers, and the second group occurred in the 30–40 cm layer. The first group contained lower relative abundances of Betaproteobacteria, Deltaproteobacteria, Actinobacteria and Firmicutes and higher relative abundances of Bacteroidetes, Gemmatimonadetes, Gammaproteobacteria, Chloroflexi, Acidobacteria and Alphaproteobacteria. The second group had higher relative abundances of Betaproteobacteria, Deltaproteobacteria, Actinobacteria and Firmicutes, but lower abundances of the other phyla (Fig. 3). According to the changes in the relative bacterial abundances with sediment depth, the dominant phyla/classes could be clustered into two categories: one category included Betaproteobacteria and Deltaproteobacteria, the relative abundances of which showed a consistent variation with depth and peaked in the 30–40 cm layer. Another category included Gemmatimonadetes, Acidobacteria, Gammaproteobacteria, Chloroflexi, Alphaproteobacteria and Bacteroidetes, the relative abundances of which also varied similarly with sediment depth. There was a significant, negative correlation between these two categories of phyla (*p* < 0.05) in the sediment profile (Table S3).

### Relationship between bacterial community composition and penguin activity

**Figure 4:**
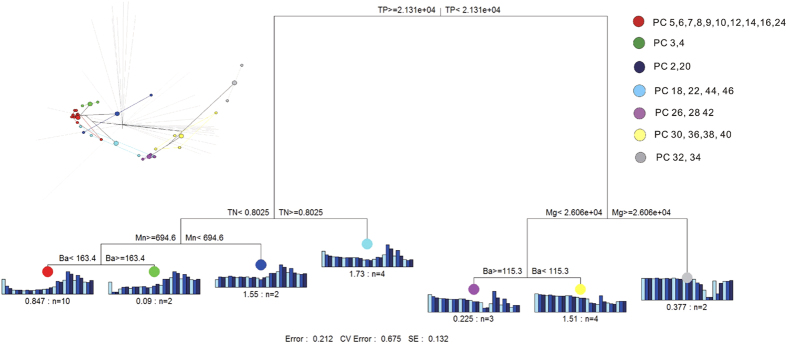
Multivariate Regression Trees (MRT) and corresponding principal components analysis (PCA) plot, indicating the geochemical constraints on the bacterial community. PC represents the respective layer (depth; cm) of the sediment profile.

The multivariate regression trees and corresponding principal components analysis indicate that the bacterial community composition in the 1–30 cm and 40–50 cm sediment layers was predominantly affected by the biogeochemical parameters related to increases in penguin activity (i.e., TP, TN, Ba, Sr, Cu and Zn; Fig. 4); in contrast, the bacterial composition in the 30–40 cm sediment layers was affected by the inputs of Fe, Mn, Mg and Al (i.e. non-biogenic elements, not related to penguin activity; Fig. 4). The Partial Mantel test showed that the changes in the bacterial community composition down the sediment profile were significantly correlated with the biogeochemical factors related to penguin activity (r = 0.23; *p* = 0.002), while there were no significant correlations with the non-biogenic elements (Fe, Mn, Mg and Al; Table 1). This therefore indicates that the overall bacterial community composition was controlled by biogeochemical factors that were related to historical penguin activity.

The relative abundances of Betaproteobacteria and Deltaproteobacteria were negatively correlated with the concentrations of the typical elements of penguin guano (TP, Sr, Ba, Cu and Zn) as well as TC and TN, and positively correlated with concentrations of Fe, Mn, Al and Mg (Table 2). Therefore the relative abundances of Betaproteobacteria and Deltaproteobacteria might be affected by inputs of Fe, Mn, Al and Mg into the lake sediments. The relative abundances of Gemmatimonadetes, Acidobacteria, Gammaproteobacteria, Chloroflexi, Alphaproteobacteria and Bacteroidetes were significantly, or weakly, positively correlated with the concentrations of TP, Sr, Ba, Cu, Zn, TC and TN, and negatively correlated with the concentrations of Fe, Mn, Al and Mg (Table 2); this further confirms that the relative abundances of these phyla were controlled by the amount of penguin guano input into the lake, that is, the intensity of historical penguin activity.

**Figure 5:**
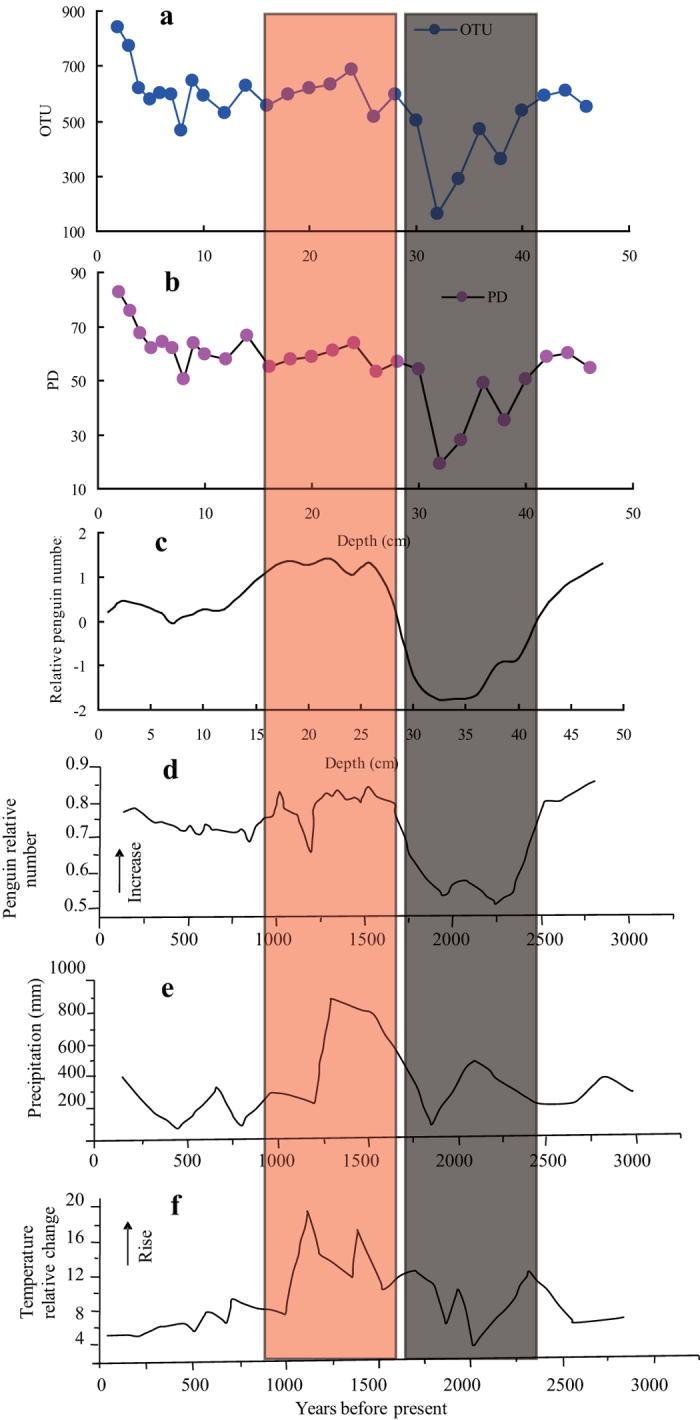
Correlation between the microbial community richness (a) and Faith’s phylogenetic diversity (b) in the ornithogenic sediments of Lake Y2, with the historical penguin population (c,d) and changes in the climate (e,f) in maritime Antarctica, over millennia. Note: the data for figure d are from Sun *et al.* (2000)^21^, and the data for figures e and f are from Zhao (1991)^5^ and Xie (2001)^71^, respectively.

The historical penguin population record around Lake Y2 was obtained from a Q-mode factor analysis and our findings were consistent with the results reported by Sun *et al.* (2000)^21^ (Fig. 5). That is, the penguin population began to decline 3,000 yr before the present (BP) and was lowest at 1,800–2,300 yr BP. After this the population increased, peaking between 1,400 and 1,800 yr BP. Interestingly, the lowest relative abundances of the bacterial phyla were found in the sediment layers corresponding to the lowest penguin population, at 1,800–2,300 yr BP, and the relative abundances of the bacterial phyla greatly increased in the sediment layers that corresponded to the peak of the penguin population, between 1,400 and 1,800 yr BP (Fig. S1). The bacterial community composition through the sediment profile showed that changes in historical penguin activity have resulted in depth-related dramatic shifts of the bacterial community.

### Relationships between bacterial richness and diversity and penguin activity

**Figure 6:**
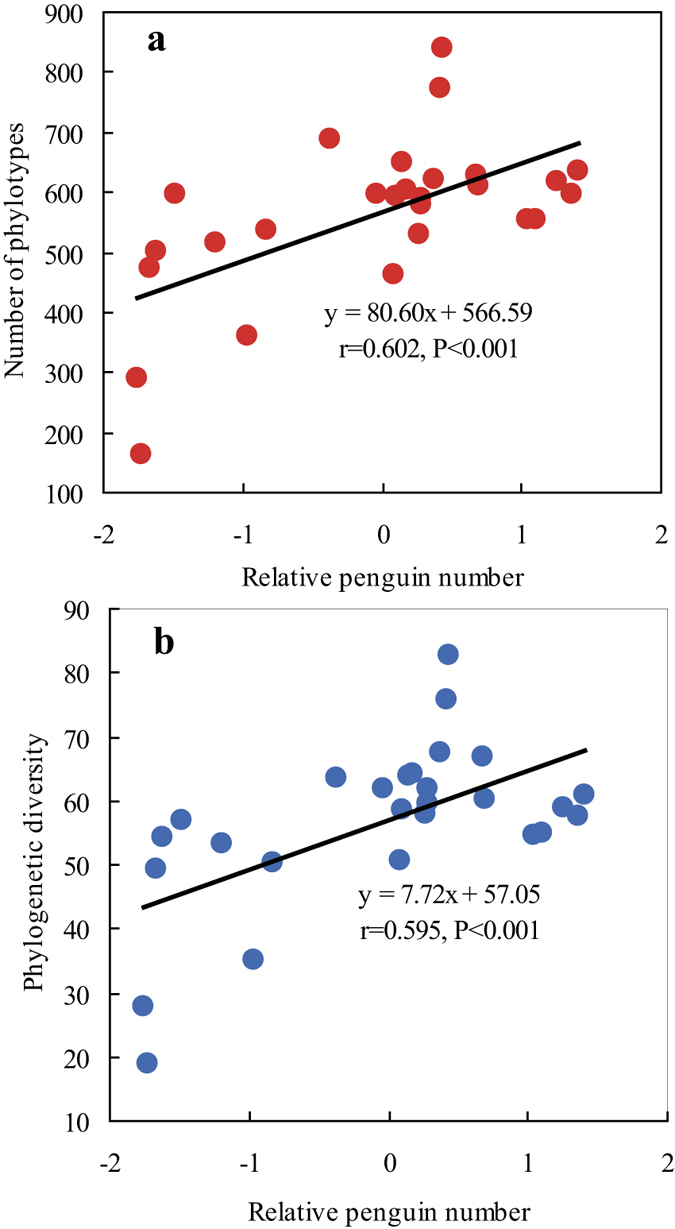
The linear relationship between the relative penguin number and both bacterial OTU richness and phylogenetic diversity. The bacterial communities from each sediment layer were randomly sampled (2,000 sequences); individual points represent the samples at different sediment depths through the sediment profile.

In terms of both phylotype richness (i.e. number of OTUs) and phylogenetic diversity (PD), which were calculated from 2,000 randomly selected sequences per sample, changes in the diversity of the bacterial community through the sediment profile varied consistently with the relative numbers of penguins around Lake Y2, except in the top 5 cm layers (Fig. 5). High bacterial phylotype richness and phylogenetic diversity occurred in the sediments corresponding to the period when the penguin population peaked between 1,400 and 1,800 yr BP^21^. The lowest bacterial diversity was found in the 30–40 cm sediments, which originate from when the penguin population was at its lowest level around 1,800–2,300 yr BP^21^, but with increasing depth, both the number of OTUs and PD returned to normal levels. Significant positive correlations (*p* < 0.01) were found between sediment bacterial phylotype richness and each of the typical elements of penguin guano (TP, Sr, Ba, Cu and Zn) as well as TC and TN; significant negative correlations were found with Fe, Mn, Mg and Al (*p* < 0.01; Table 3 and Fig. S3). Similarly, bacterial phylogenetic diversity was positively correlated (*p* < 0.01) with sediment concentrations of TP, Sr, Ba, Cu, Zn and TC, TN, and negatively correlated (*p* < 0.01) with Fe, Mn, Mg and Al (Figs S4 and S5). The bacterial phylotype richness and phylogenetic diversity through the sediment profile were also significantly positive correlated with the relative penguin number (RPN; *p* = 0.001; Fig. 6). Therefore historical penguin activity around Lake Y2 has significantly altered the microbial diversity in the sediments, even over millennia.

The bacterial diversity through the sediment profile declined in the sediment layer that corresponded to the decline in the penguin population at approximately 2,800 yr BP, in response to the glacial advances on King George Island^38^. The lower bacterial diversity that occurred in the sediment corresponding to around 1,800–2,300 yr BP was in accordance with lower temperatures and precipitation^39^, whereas higher bacterial diversity corresponded to a moister, and presumably warmer climate, between 1800 and 1400 BP^21^. Accompanying the fluctuations in the penguin population, climate change was probably responsible for the changes in bacterial diversity that occurred in the ornithogenic sediment profile of Lake Y2 (Fig. 5). Therefore, the depth-related variations of the microbial community in the sediments, and changes in the penguin population, might correspond to times of climate change in maritime Antarctica, over millennia.

## Discussion

A number of studies have researched the microbial community structure in Antarctic lake sediments, based on 16S rRNA genes, PCR-DGGE or stripe cloning sequencing analysis^11^,^12^,^13^,^35^,^37^,^40^,^41^,^42^; unfortunately, using these methods the microbial community composition in these studies could not be accurately described within each layer of the sediment profiles, due to relatively low taxonomic resolution. In this study, the ornithogenic sediment profile collected provided an opportunity to examine the potential effects of historical penguin activity on bacterial community composition, diversity and succession in the sediments; detailed information about the bacterial community structure could be elucidated from the ornithogenic sediments, based on the 454 sequencing analyses.

Our results indicate that historical penguin activity has significantly impacted the vertical distribution pattern of bacterial community composition in the sediments, through the deposition of their guano. In the Lake Y2 sediments, Proteobacteria, Actinobacteria, Gemmatimonadetes, Bacteroidetes, Acidobacteria and Chloroflexi were found in high relative abundances (Fig. 2 and Fig. S2). Although most of the sequences had high similarities to those of uncultured microorganisms retrieved from lake sediments^13^,^43^, soils^35^,^44^,^45^,^46^,^47^, and ice^48^ in Antarctica, the percentages of each class within the phylum Proteobacteria, and of other phyla, varied greatly with depth, which was related to historical penguin activity (Fig. S1). Historical penguin activity had altered the sediment biogeochemical properties at each depth measured, and these properties significantly co-varied with changes in the bacterial community composition in the sediment profile (Fig. 1 and Fig. S1). In maritime Antarctica, the deposition of penguin guano is an important source of nutrients for soils and sediments in local terrestrial and aquatic ecosystems^20^,^21^,^25^. Generally the mean concentrations of TN, TP, Sr, Ba, Cu and Zn have previously been found to be significantly higher in ornithogenic than non-ornithogenic soils and sediments; this assemblage of elements, including P, Sr, Ba, Cu, Zn, is an important geochemical characteristic of ornithogenic lake sediments in maritime Antarctica^21^,^24^,^25^,^27^. Krill is the main bulk component of penguin diets in the study area, and the effects of the diets on the relative levels of these elements are expected to be insignificant^21^,^36^. In the ornithogenic sediments, the deposition amount of penguin guano reflected the relative penguin population size during the historical period. The levels for these typical elements in the sediments were controlled by the deposition amount of penguin guano, i.e. the relative penguin population size^21^,^24^,^25^. The relative abundances of dominant phyla, or classes (Gammaproteobacteria, Gemmatimonadetes, Acidobacteria, Chloroflexi, Alphaproteobacteria, Actinobacteria and Bacteroidetes), were positively correlated with sediment TP, Sr, Ba, Cu, Zn, TC and TN (Table 2), indicating that historical penguin activity (i.e. the input amount of penguin guano into the sediments) was the primary factor that affected the bacterial abundances (Fig. 4). The Partial Mantel test further confirmed that bacterial community composition was significantly correlated with the biogeochemical factors related to historical penguin activity (Table 1). The deposition of a large amount of penguin guano could provide extra substrate for sediment bacteria, which might increase the bacterial abundance^28^. Similarly, it was found that the abundances of chitinolytic bacteria in sediments were significantly correlated with TP and TOC content; this suggests a historical connection between the abundance of chitinolytic bacteria and the amount of penguin guano deposited on the sediments^37^.

In the 30–40 cm sediment layers, Betaproteobacteria and Deltaproteobacteria showed an extremely high relative abundance, which coincided with lower penguin activity and increases in the concentrations of Fe, Mn, Mg and Al (Fig. 1 and Fig. S1). Some dissimilatory iron-reducing microorganisms (DIRM) belong to the classes Betaproteobacteria and Deltaproteobacteria, thus the enrichment of Fe and Mn and decrease in the nutrients sourced from penguin guano, might be responsible for relatively high abundances of Betaproteobacteria and Deltaproteobacteria^49^. Other dominant phyla decreased in their abundances in sediment layers that corresponded to lower penguin activity. Overall, we consider that historical penguin activity caused the large variability in the biogeochemical properties of the sediment, through the inputs of their guano, and thus influenced the vertical distribution pattern of bacterial abundance in the ornithogenic sediments.

The bacterial diversity in the sediments collected from Lake Y2 also varied greatly down the vertical profile, in response to changes in the historical penguin population (Fig. 5). The significant correlations found between phylotype richness (i.e. the number of OTUs), phylogenetic diversity and the relative penguin number (RPN) further confirmed that historical penguin activity has had an important effect on the sediment bacterial diversity (Fig. 6). In the Ross Sea region, the bacteria found in mineral soils were dominated by a few bacterial phylotypes, and the soil bacterial community had a relatively low diversity, whereas ornithogenic soils have generally had a high soil bacterial diversity when close to penguin colonies^32^,^33^. In the Antarctic Dry Valleys, it seems that abiotic physical modifications, such as stabilizing temperatures, elevating relative humidity and reducing ultraviolet exposure, associated with the presence of a seal carcass, rather than nutrient or biological inputs, led to changes in a microbial community^50^. Soil samples collected from eight locations around the Casey Station indicated that higher bacterial diversity occurred in soils located next to a penguin rookery^51^. Ma *et al.* (2013)^35^ observed a correlation between bacterial abundance and diversity, and the quantity of penguin guano within penguin colony soils, in east Antarctica. Investigations of the faeces of gulls have also suggested that the input of animal excreta could change soil bacterial diversity^52^. In this study, effects of penguin activity on the bacterial diversity in the ornithogenic sediment layers were similar to those reported above in similar substrates^32^,^33^,^35^,^51^. Therefore, penguin activity could indirectly affect sediment bacterial diversity, by altering the sediment biogeochemical properties.

It was expected that higher phylotype richness (i.e. number of OTUs) and phylogenetic diversity would be present in the top 5 cm of the ornithogenic sediments. The bacterial distribution in surface sediments may be complex, due to rapid changes in physicochemical and geochemical factors^53^, and active bioturbation, which introduces fresh nutrients from the lakewater–sediment interface and physically redistributes the bacterial community structure^11^,^40^. Differences in the community diversity did not correlate with the chemical properties measured in the top 5 cm of the sediments; a significant portion of the microbiota may have been at least partially responding to the fluctuating conditions, rather than primarily responding to the penguin guano inputs^53^.

The lowest bacterial diversity was found in the 30–40 cm layers of the sediment, which corresponded to the lowest levels of the penguin population. In these layers, only a few bacterial species dominated the community. This was probably due to strong competitive exclusion by the dominant phyla Betaproteobacteria and Deltaproteobacteria^54^,^55^.

Factors that influence the structure of microbial communities, including the availability of nutrients, pH and texture, can vary considerably with the depth of a soil or sediment^56^. The redox potential in different depth sediments might be a strong driving factor for the bacterial community structure. The deposition of penguin guano could provide abundant nutrients and extra substrate for sediment bacteria, and further change the sediment pH, texture and redox potential (Fig. 1). This might lead to the variation in sediment bacterial community structure. Sulfate-reducing Deltaproteobacteria were abundant in the 30–40 cm sediment layers, whereas they showed a low relative abundance in other sediment layers, indicating that low redox potential occurred in the 30–40 cm sediments, and these obligate anaerobic bacteria might thrive in anaerobic microniches^12^. In this study, penguins would have not only contributed abiotic components to the lake sediments but also biotic components, such as bacterial inoculum. It appeared that the vertical distribution of bacterial diversity was associated with sediment biogeochemical processes, related to the historical penguin activity (i.e., the supply of nutrients TP, TN, and TC and other typical components of penguin guano, including Sr, Ba, Cu, Zn). Furthermore our data showed that significant changes in the structure of the Antarctic ornithogenic sediment microbial communities were associated with historical penguin activity that took place within the timeframe of millennia, and these changes in penguin activity seem to dramatically affect biodiversity today. In addition, genomic DNA retrieved from the ornithogenic sediment profile PC might include both ancient and current microbial information as the DNA could be adsorbed by the minerals, and preserved over long time period under the extreme environmental conditions in Antarctica^13^,^35^,^37^. Therefore our results might not only represent the current bacterial community structure in the sediment profile, but also partially reflect the bacterial community structure when the ornithogenic sediments formed.

In coastal Antarctica, penguins are considered to be ideal bio-indicators of climate change^57^. In our study area, the penguin population was lowest at 1,800–2,300 yr BP, a period of low temperatures, and the population increased to its peak between 1,400 and 1,800 yr BP, corresponding almost exactly to a relatively warm period^21^,^38^,^39^. Therefore past changes in the climate could have had an impact on the historical penguin population, and in turn these changes currently influence the bacterial abundance and diversity down the ornithogenic sediment profile (Fig. 5). The bacterial communities in the 30–40 cm layers of the sediment had the lowest species diversity, and these layers correspond to a drier and colder period, between 2300 and 1800 BP. Higher bacterial diversity was found in the 15–30 cm layers of sediment, which corresponds to a moister, and presumably warmer climate, between 1800 and 1400 BP^21^.

Any change in climate conditions will be accompanied by multiple environmental stressors to the bacterial communities in the lake sediments, including modifications to temperature as well as changes in the nutrient inputs from penguins. Our results have shown that indigenous microbial communities of ornithogenic sediments can experience long-term and sustained structural alterations in response to changes in the inputs of historical penguin guano and the climate. It seems that the microbial community and penguin populations might almost simultaneously correspond to climate change within maritime Antarctica, over millennia. The variations of the bacterial community composition in the ornithogenic sediment profile could indirectly reflect fluctuations in the penguin population and climate change. These results provide support for our three hypotheses.

In conclusion, we have successfully addressed three important questions about the microbial ecology through the profile of ornithogenic sediments in maritime Antarctica. Our results have demonstrated that historical penguin activity has significantly impacted the vertical distribution pattern of bacterial community composition and diversity, by altering the sediment biogeochemical processes. Furthermore, significant changes in the structure of the Antarctic ornithogenic sediment microbial communities, associated with historical penguin activity, can take place within the timeframe of millennia, and past increases in penguin activity seem to have led to dramatic increases in microbial biodiversity within the relevant sediment layers. Together, our results suggest that changes to the climate could have had an impact on the historical penguin population and, in turn, influenced the bacterial abundance and diversity down the ornithogenic sediment profile. The changes in the microbial diversity within the sediment profile could provide baseline information to predict penguin populations in the future, as well as the regional response of the Antarctic to climate change.

## Materials and Methods

### Sample collection

The study area was located on Ardley Island (62°13′S, 58°56′W; 2.0 km length and 1.5 km width), just off the southwest point of King George Island, in maritime Antarctica. There exists a large number of breeding penguins on the island, including Gentoo penguins (*Pygoscelis papua*), Adélie penguins (*Pygoscelis adeliae*) and Chinstrap penguins (*Pygoscelis antarctica*). During the breeding period, it was estimated that penguins on Ardley Island discharge about 139 tonnes of guano; some of this guano was deposited in the lakes or ponds around the penguin colonies, transferred by ice or snowmelt water^38^. During the summer of 2011/2012, a 50 cm ornithogenic sediment core (named as PC) was collected from Lake Y2 on Ardley Island, using a clean PVC pipe with a 12 cm diameter (Fig. S6). The ornithogenic sediment rate was very slow with an average rate of about 0.02 cm yr^−1^, and the geology structure of the sediments was highly stable in this lake^21^. Therefore, the related data published in 1991–2000 could be cited to explain the results obtained from the samples collected 20 years later. The biogeochemistry of the sediment in Lake Y2 was previously shown to be strongly affected by historical penguin activity, through the deposition of their guano, by Sun *et al.* (2000)^21^. Immediately after collection, the core was sealed and stored below −20 °C until laboratory analysis. The upper 45 cm of the sediment core was divided into 45 subsamples, each consisting sediment from 1.0 cm of depth, and the bottom 46–50 cm of the core comprised the 46th subsample. The sediment subsamples were divided into two portions: one portion was stored in sterile plastic containers at −80 °C for the analysis of the bacterial community structure in the sediments, and the other portion was stored in HNO_3_-washed bottles to determine the sediment geochemical characteristics. After the sediment core was sectioned, all of the sediment analyses were performed within one month.

### General analysis of sediment geochemical characteristics

Each subsample was homogenised for the general analyses. The pH was determined in distilled water and in a 1M KCl solution (at a sediment:solution ratio of 1:3). Total carbon (TC) and total nitrogen (TN) were determined using a CNS Elemental Analyzer (Vario EL III), with a relative error of 0.1%^26^. Total phosphorus (TP) was analysed by measuring the phosphate concentration, using the ammonium molybdate spectrophotometric method, after digestion^58^. The concentrations of Sr and Ba were determined using inductively coupled plasma-atomic emission spectrometry (ICP-AES; Model Atom Scan Advantage from Thermo Elemental)^36^. The concentrations of Cu, Zn, Mn and Mg were determined using atomic absorption spectrometry (AAS novAA 400), and the concentrations of Fe and Al were measured using various wet chemical methods^36^. The elements P, Sr, Ba, Cu and Zn had previously been identified as the typical elements in penguin guano^21^.

### Sediment DNA extraction

Genomic DNA was extracted from 0.5 g of sediment from each subsample using a FastDNA^®^ SPIN Kit for soil (Bio 101, Carlsbad, CA, USA), according to the manufacturer’s instructions. The raw DNA was purified using a 0.8% (wt/vol) low melting point Agarose Gel, to obtain whole genomic DNA. The DNA bands were excised, then extracted using an Agarose Gel DNA purification kit (TaKaRa). The purified DNA was then quantified with a NanoDrop ND-1000 spectrophotometer (NanoDrop Technologies, Wilmington, DE, USA)^59^.

### 454 pyrosequencing

Bacterial 16S rRNA genes were amplified using the primer set F519 and R907^60^,^61^. The forward primer was attached to the Roche 454 ‘B’ pyrosequencing adapter and a unique 7 bp barcode; the reverse primer was attached to the Roche 454 ‘A’ sequencing adapter. Bar-code PCR amplifications were conducted in a 25 μL reaction mixture of 2 × Pre-Mix (TAKARA), 0.5 μl × 20 mM of each forward and reverse primer, 50 ng of DNA and then made up to 50 μL with double distilled water. Each sample was amplified in triplicate, with 30 cycles of: 94 °C for 30 s, 55 °C for 30 s and 72 °C for 30 s, and then a final extension cycle at 72 °C for 10 min. The PCR products were pooled together and purified using an Agarose Gel DNA purification kit (TaKaRa). An equal amount of PCR product of each triplicate, determined qualitatively using a bioanalyzer (Agilent 2100) and quantitatively using the NanoDrop, was pooled into a single tube. The pooled sample was run on a Roche FLX 454 pyrosequencing machine (Roche Diagnostics Corporation, Branford, CT, USA), which produced reads from the forward direction F519.

### Data processing

Data were processed using the Quantitative Insight Into Microbial Ecology (QIIME) pipeline^62^. Briefly, bacterial sequences with the same barcode were assigned to the same sample after denoising with a denoiser v. 0.91^63^. The barcode and primer sequences were removed, so only the target fragment after the proximal PCR primer was included for further analysis. Bacterial phylotypes were identified using uclust^64^ and assigned to operational taxonomic units (OTUs, at the 97% sequence similarity level). A representative sequence from each phylotype was aligned using PyNAST^65^,^66^. The taxonomic identity of each phylotype was classified using the Ribosomal Database Project (RDP)^67^. To correct for survey effort, a total of 2000 sequences per sample were randomly selected for the comparison of the relative differences between the samples.

### Statistical analyses

Phylogenetic diversity (PD) was estimated by Faith’s index^68^, which provides an integrated index of the phylogenetic breadth across taxonomic levels. The relationships between the taxonomic diversity and geochemical characteristics of each sediment layer were tested using linear regression analyses in SPSS 20.0 for Windows. In order to identify the geochemical factors associated with changes in the bacterial community composition, correlation analyses between the relative abundance of dominant phyla and the geochemical factors were conducted in SPSS 20.0. A heatmap diagram of the 10 most dominant bacterial phyla among the 27 sediment layers was generated in Cluster 3.0 and Treeview^69^. In addition, we created Multivariate Regression Trees (MRT) and a corresponding principal components analysis (PCA) plot, to show the relative constraints of the key environmental variables on the bacterial community, using the “mvpart” package in R^70^. The Partial Mantel test was used to explain the correlations between the bacterial community composition and the geochemical factors and their relationship with historical penguin activity.

A Q-factor analysis was performed to explore the relationships between the bacterial community structure and the amount of guano deposition, using SPSS 20.0 and Microsoft Excel 2003. In Lake Y2, P, Sr, Ba, Cu and Zn had been identified as the typical elements of penguin guano^21^. Therefore the concentrations of TP, Sr, Ba, Cu and Zn in the sediments were used for the Q-factor analysis to indicate the relative amount of penguin guano input (i.e. the relative size of the penguin population) over the historical period represented by the sediment core. Radiocarbon dating of Antarctic ornithogenic sediments and marine fauna is complicated by the marine reservoir effect, which results from the unusual radiocarbon content of the Southern Ocean coastal waters^50^. The radiocarbon ages for the ornithogenic sediments in Lake Y2, and the historical penguin population records, are shown in Sun *et al.* (2000)^21^. The ages of the sediment from the PC core were obtained according to the contrasts between the penguin population records in Sun *et al.* (2000)^21^ and this study.

## Additional Information

**How to cite this article**: Zhu, R. *et al.* Bacterial diversity is strongly associated with historical penguin activity in an Antarctic lake sediment profile. *Sci. Rep.* **5**, 17231; doi: 10.1038/srep17231 (2015).

## Supplementary Material

Supplementary Information

## Figures and Tables

**Table 1:** The correlations (r) and significance (*p*), determined by a Partial Mantel Test, between the bacterial community composition and biogeochemical factors in the sediment profile of Lake Y2.

Type	Factor 1	Factor 2
r	**0.228**	0.028
*p*	**0.002**	0.338

Note: Factor 1 comprises the geochemical parameters related to historical penguin activity, including the concentrations of typical elements of penguin guano (TP, Sr, Ba, Cu and Zn) as well as TN and TC in the sediments. Factor 2 consists of the non-biogenic parameters, which are not related to penguin activity, including the concentrations of Fe, Mn, Mg and Al in the sediments

**Table 2:** The correlations between bacterial abundance and geochemical factors in sediments from Lake Y2, in maritime Antarctica.

Geochemicalfactors	Actinobacteria	Gemmatimonadetes	Bacteroidetes	Gammaproteobacteria	Acidobacteria	Chloroflexi	Alphaproteobacteria	Deltaproteobacteria	Betaproteobacteria
TC	**0.132**	0.615**	**0.155**	**0.358**	0.422*	0.511**	0.728**	−0.332	−0.670**
TN	**0.166**	0.556**	**0.194**	0.381*	**0.347**	0.451*	0.623**	−0.310	−0.611**
TP	**0.066**	0.619**	**0.094**	0.407*	0.495**	0.523**	0.773**	−0.338	−0.690**
pH	**0.058**	0.443*	**0.071**	**0.044**	0.426*	0.470*	0.513**	−0.036	−0.487**
Cu	**0.108**	0.604**	**0.158**	0.394*	**0.366**	0.480**	0.752**	−0.301	−0.655**
Zn	**0.143**	0.614**	**0.157**	0.389*	0.396*	0.476*	0.748**	−0.319	−0.676**
Sr	**0.082**	0.637**	**0.163**	0.409*	0.390*	0.490**	0.759**	−0.331	−0.680**
Ba	**0.150**	0.654**	**0.073**	**0.231**	0.554**	0.515**	0.682**	−0.298	−0.693**
Fe	−0.116	−0.643**	−0.142	−0.337	−0.481**	−0.529**	−0.767**	**0.293**	0.710**
Mn	−0.159	−0.684**	−0.100	−0.322	−0.565**	−0.574**	−0.790**	**0.300**	0.756**
Mg	−0.112	−0.621**	−0.121	−0.350	−0.469*	−0.535**	−0.781**	**0.284**	0.699**
Al	−0.148	−0.482**	−0.029	−0.332	−0.271	−0.411*	−0.652**	**0.136**	0.547**

Note: **Correlation is significant at the 0.01 level (2-tailed), and *Correlation is significant at the 0.05 level (2-tailed)

**Table 3:** The correlations between bacterial OTU richness, phylogenetic diversity (PD) and biogeochemical factors in the sediment profile of Lake Y2, on Ardley Island, in maritime Antarctica.

Geochemical factors	OTU		PD	
	r	P	r	P
TC	0.575**	**0.002**	0.570**	**0.002**
TN	0.517**	**0.006**	0.488**	**0.010**
TP	0.669**	**0.001**	0.672**	**0.001**
pH	0.528**	**0.005**	0.636**	**0.001**
Cu	0.562**	**0.002**	0.541**	**0.004**
Zn	0.564**	**0.002**	0.550**	**0.003**
Sr	0.573**	**0.002**	0.560**	**0.002**
Ba	0.616**	**0.001**	0.649**	**0.001**
RPN	0.602**	**0.001**	0.595**	**0.001**
Fe	−0.628**	0.001	−0.644**	0.001
Mn	−0.682**	0.001	−0.694**	0.001
Mg	−0.634**	0.001	−0.645**	0.001
Al	−0.568**	0.001	−0.545**	0.003

Notes: *Correlation is significant at the 0.05 level (2-tailed); **correlation is significant at the 0.01 level (2-tailed). RPN = relative penguin numbers during the historical period, based on the Q-factor analysis.

## References

[b1] Ellis-Evans J. C. Microbial diversity and function in Antarctic freshwater ecosystems. Biodivers. Conserv. 5, 1395–1431 (1996).

[b2] Shivaji S. *et al.* Vertical distribution of bacteria in a lake sediment from Antarctica by culture-independent and culture-dependent approaches. Res. Microbiol. 162, 191–203 (2011). 21126578 10.1016/j.resmic.2010.09.020

[b3] Priscu J. C. *et al.* Perennial Antarctic lake ice: An oasis for life in a polar desert. Science 280, 2095–2098 (1998). 9641910 10.1126/science.280.5372.2095

[b4] Jurgens G. *et al.* Identification of novel Archaea in bacterioplankton of a boreal forest lake by phylogenetic analysis and fluorescent *in situ* hybridization. FEMS Microbiol. Ecol. 34, 45–56 (2000). 11053735 10.1111/j.1574-6941.2000.tb00753.x

[b5] Zhao J. L. In The Characteristics of the Modern Environmental Geochemistry and Natural Environmental Evolution in the Region of Antarctic Great Wall Station [ Zhao J. L. (ed.)][31–92] (Science Press, Beijing, 1991).

[b6] Hodgson D. A. *et al.* Were the Larsemann Hills ice-free through the Last Glacial Maximum? Antarct. Sci. 13, 440–454 (2001).

[b7] Spring S., Schulze R., Overmann J. & Schleifer K. H. Identification and characterization of ecologically significant prokaryotes in the sediment of freshwater lakes: molecular and cultivation studies. FEMS Microbiol. Rev. 24, 573–590 (2000). 11077151 10.1111/j.1574-6976.2000.tb00559.x

[b8] Urakawa H., Yoshida T., Nishimura M. & Ohwada K. Characterization of depth-related population variation in microbial communities of a coastal marine sediment using 16S rDNA-based approaches and quinone profiling. Environ. Microbiol. 2, 542–554 (2000). 11233162 10.1046/j.1462-2920.2000.00137.x

[b9] Inagaki F., Sakihama Y., Inoue A., Kato C. & Horikoshi K. Molecular phylogenetic analyses of reverse-transcribed bacterial rRNA obtained from deep-sea cold seep sediments. Environ. Microbiol. 4, 277–286 (2002). 12030853 10.1046/j.1462-2920.2002.00294.x

[b10] Bowman J. P. & McCuaig R. D. Biodiversity, community structural shifts, and biogeography of prokaryotes within Antarctic continental shelf sediment. Appl. Environ. Microbiol. 69, 2463–2483 (2003). 12732511 10.1128/AEM.69.5.2463-2483.2003 PMC154503

[b11] Bowman J. P., McCammon S. A., Gibson J. A. E., Robertson L. & Nichols P. D. Prokaryotic metabolic activity and community structure in Antarctic continental shelf sediments. Appl. Environ. Microbiol. 69, 2448–2462 (2003). 12732510 10.1128/AEM.69.5.2448-2462.2003 PMC154502

[b12] Sjoling S. & Cowan D. A. High 16S rDNA bacterial diversity in glacial meltwater lake sediment, Bratina Island, Antarctica. Extremophiles 7, 275–282 (2003). 12910387 10.1007/s00792-003-0321-z

[b13] Li S. K., Xiao X., Yin X. B. & Wang F. P. Bacterial community along a historic lake sediment core of Ardley Island, west Antarctica. Extremophiles 10, 461–467 (2006). 16715185 10.1007/s00792-006-0523-2

[b14] Ye W. *et al.* The vertical distribution of bacterial and archaeal communities in the water and sediment of Lake Taihu. FEMS Microbiol. Ecol. 70, 263–276 (2009). 10.1111/j.1574-6941.2009.00761.x 19744240

[b15] Ribeiro H., Mucha A. P., Almeida C. M. R. & Bordalo A. A. Bacterial community response to petroleum contamination and nutrient addition in sediments from a temperate salt marsh. Sci. Total Environ. 458, 568–576 (2013). 23707865 10.1016/j.scitotenv.2013.04.015

[b16] Costello A. M., Auman A. J., Macalady J. L., Scow K. M. & Lidstrom M. E. Estimation of methanotroph abundance in a freshwater lake sediment. Environ. Microbiol. 4, 443–450 (2002). 12153585 10.1046/j.1462-2920.2002.00318.x

[b17] Gregory L. G., Bond P. L., Richardson D. J. & Spiro S. Characterization of a nitrate-respiring bacterial community using the nitrate reductase gene (narG) as a functional marker. Microbiol.-SGM 149, 229–237 (2003). 10.1099/mic.0.25849-0 12576596

[b18] Purdy K. J., Nedwell D. B. & Embley T. M. Analysis of the sulfate-reducing bacterial and methanogenic archaeal populations in contrasting Antarctic sediments. Appl. Environ. Microbiol. 69, 3181–3191 (2003). 12788715 10.1128/AEM.69.6.3181-3191.2003 PMC161550

[b19] Ugolini F. C. In Antarctic Terrestrial Biology [ Ugolini F. C. (ed.)][181–193](American Geophysical Union, 2013).

[b20] Tatur A., Myrcha A. & Niegodzisz J. Formation of abandoned penguin rookery ecosystems in the maritime Antarctic. Polar Biol. 17, 405–417 (1997).

[b21] Sun L. G., Xie Z. Q. & Zhao J. L. A 3,000-year record of penguin populations. Nature 407, 858–858 (2000). 11057656 10.1038/35038163

[b22] Hofstee E. H., Balks M. R., Petchey F. & Campbell D. I. Soils of Seabee Hook, Cape Hallett, northern Victoria Land, Antarctica. Antarct. Sci. 18, 473–486 (2006).

[b23] Beyer L. *et al.* Soil organic matter of suggested spodic horizons in relic ornithogenic soils of coastal continental Antarctica (Casey Station, Wilkes Land) in comparison with that of spodic soil horizons in Germany. Soil Sci. 162, 518–527 (1997).

[b24] Tatur A. In *Geoecology of Antarctic Ice-Free Coastal Landscapes*, Vol. 154 Ecological Studies [ Beyer L. *et al.* (ed.)][161–184] (Springer Berlin Heidelberg, 2002).

[b25] Simas F. N. B. *et al.* Ornithogenic cryosols from Maritime Antarctica: Phosphatization as a soil forming process. Geoderma 138, 191–203 (2007).

[b26] Zhu R. B., Sun J. J., Liu Y. S., Gong Z. J. & Sun L. G. Potential ammonia emissions from penguin guano, ornithogenic soils and seal colony soils in coastal Antarctica: effects of freezing-thawing cycles and selected environmental variables. Antarct. Sci. 23, 78–92 (2011).

[b27] Bölter M., Kandeler E., Pietr S. & Seppelt R. In Geoecology of Antarctic Ice-Free Coastal Landscapes [ Bölter M. *et al.* . (ed.)][189–214] (Springer Berlin Heidelberg, 2002).

[b28] Zdanowski M. K., Zmuda M. J. & Zwolska W. Bacterial role in the decomposition of marine-derived material (penguin guano) in the terrestrial maritime Antarctic. Soil Biol. Biochem. 37, 581–595 (2005).

[b29] Yergeau E., Newsham K. K., Pearce D. A. & Kowalchuk G. A. Patterns of bacterial diversity across a range of Antarctic terrestrial habitats. Environ. Microbiol. 9, 2670–2682 (2007). 17922752 10.1111/j.1462-2920.2007.01379.x

[b30] Zhu R. B., Liu Y. S. W., Xu H., Ma D. W. & Jiang S. Marine animals significantly increase tundra N_2_O and CH_4_ emissions in maritime Antarctica. J. Geophys. Res-Biogeosci. 118, 1773–1792 (2013).

[b31] Zhu R. B., Bao T., Wang Q., Xu H. & Liu Y. S. Summertime CO_2_ fluxes and ecosystem respiration from marine animal colony tundra in maritime Antarctica. Atmos. Environ. 98, 190–201 (2014).

[b32] Aislabie J. M., Jordan S. & Barker G. M. Relation between soil classification and bacterial diversity in soils of the Ross Sea region, Antarctica. Geoderma 144, 9–20 (2008).

[b33] Aislabie J. *et al.* Bacterial diversity associated with ornithogenic soil of the Ross Sea region, Antarctica. Can. J. Microbiol. 55, 21–36 (2009). 19190698 10.1139/W08-126

[b34] Chong C. *et al.* Environmental influences on bacterial diversity of soils on Signy Island, maritime Antarctic. Polar Biol. 32, 1571–1582 (2009).

[b35] Ma D. W. *et al.* *Ex-situ* enzyme activity and bacterial community diversity through soil depth profiles in penguin and seal colonies on Vestfold Hills, East Antarctica. Polar Biol. 36, 1347–1361 (2013).

[b36] Sun L. G. *et al.* A geochemical method for the reconstruction of the occupation history of a penguin colony in the maritime Antarctic. Polar Biol. 27, 670–678 (2004).

[b37] Xiao X. *et al.* Chitinase genes in lake sediments of Ardley Island, Antarctica. Applied And Environ. Microbiol. 71, 7904–7909 (2005). 10.1128/AEM.71.12.7904-7909.2005 PMC1317360 16332766

[b38] Sun L. G. & Xie Z. Q. Relics: penguin population programs. Sci prog. 84, 31–44 (2001). 11382136 10.3184/003685001783239078 PMC10361192

[b39] Clapperton C. M., Sugden D. E., Birnie J. & Wilson M. J. Late-glacial and Holocene glacier fluctuations and environmental change on South Georgia, Southern Ocean. Quaternary Res. 31, 210–228 (1989).

[b40] Turley C. Bacteria in the cold deep-sea benthic boundary layer and sediment-water interface of the NE Atlantic. FEMS Microbiol. Ecol. 33, 89–99 (2000). 10967208 10.1111/j.1574-6941.2000.tb00731.x

[b41] Bowman J. P., Rea S. M., McCammon S. A. & McMeekin T. A. Diversity and community structure within anoxic sediment from marine salinity meromictic lakes and a coastal meromictic marine basin, Vestfold Hills, Eastern Antarctica. Environ. Microbiol. 2, 227–237 (2000). 11220308 10.1046/j.1462-2920.2000.00097.x

[b42] Wang P., Xiao X. & Wang F. P. Phylogenetic analysis of Archaea in the deep-sea sediments of west Pacific Warm Pool. Extremophiles 9, 209–217 (2005). 15761691 10.1007/s00792-005-0436-5

[b43] Pearce D. A., van der Gast C. J., Lawley B. & Ellis-Evans J. C. Bacterioplankton community diversity in a maritime Antarctic lake, determined by culture-dependent and culture-independent techniques. FEMS Microbiol. Ecol. 45, 59–70 (2003). 19719607 10.1016/S0168-6496(03)00110-7

[b44] Shivaji S., Reddy G. S. N., Aduri R. P., Kutty R. & Ravenschlag K. Bacterial diversity of a soil sample from Schirmacher Oasis, Antarctica. Cell. Mol. Biol. 50, 525–536 (2004). 15559969

[b45] Cary S. C., McDonald I. R., Barrett J. E. & Cowan D. A. On the rocks: the microbiology of Antarctic Dry Valley soils. Nat. Rev. Microbiol. 8, 129–138 (2010). 20075927 10.1038/nrmicro2281

[b46] Moosvi S. A., McDonald I. R., Pearce D. A., Kelly D. P. & Wood A. P. Molecular detection and isolation from Antarctica of methylotrophic bacteria able to grow with methylated sulfur compounds. Syst. Appl. Microbiol. 28, 541–554 (2005). 16104352 10.1016/j.syapm.2005.03.002

[b47] Teixeira L. C. R. S. *et al.* Bacterial diversity in rhizosphere soil from Antarctic vascular plants of Admiralty Bay, maritime Antarctica. ISME J. 4, 989–1001 (2010). 20357834 10.1038/ismej.2010.35

[b48] Gordon D. A., Priscu J. & Giovannoni S. Origin and phylogeny of microbes living in permanent Antarctic lake ice. Microb. Ecol. 39, 197–202 (2000). 12035096 10.1007/s002480000016

[b49] Emerson D., Fleming E. J. & McBeth J. M. Iron-oxidizing bacteria: an environmental and genomic perspective. Annu. Rev. Microbiol. 64, 561–583 (2010). 20565252 10.1146/annurev.micro.112408.134208

[b50] Tiao G., Lee C. K., McDonald I. R., Cowan D. A. & Cary S. C. Rapid microbial response to the presence of an ancient relic in the Antarctic Dry Valleys. Nat. Commun. 3, 660, doi: 10.1038/ncomms1645 (2012). 22314356

[b51] Chong C. W., Tan G. Y. A., Wong R. C. S., Riddle M. J. & Tan I. K. P. DGGE fingerprinting of bacteria in soils from eight ecologically different sites around Casey Station, Antarctica. Polar Biol. 32, 853–860 (2009).

[b52] Lu J. R., Santo Domingo J. W., Lamendella R., Edge T. & Hill S. Phylogenetic diversity and molecular detection of bacteria in gull feces. Appl. Environ. Microbiol. 74, 3969–3976 (2008). 18469128 10.1128/AEM.00019-08 PMC2446513

[b53] D’Hondt S., Rutherford S. & Spivack A. J. Metabolic activity of subsurface life in deep-sea sediments. Science 295, 2067–2070 (2002). 11896277 10.1126/science.1064878

[b54] Zhou J. Z. *et al.* Spatial and resource factors influencing high microbial diversity in soil. Appl. Environ. Microbiol. 68, 326–334 (2002). 11772642 10.1128/AEM.68.1.326-334.2002 PMC126564

[b55] Tringe S. G. *et al.* Comparative metagenomics of microbial communities. Science 308, 554–557 (2005). 15845853 10.1126/science.1107851

[b56] Hansel C. M., Fendorf S., Jardine P. M. & Francis C. A. Changes in bacterial and archaeal community structure and functional diversity along a geochemically variable soil profile. Appl. Environ. Microbiol. 74, 1620–1633 (2008). 18192411 10.1128/AEM.01787-07 PMC2258623

[b57] Baroni C. & Orombelli G. Abandoned penguin rookeries as holocene paleoclimatic indicators in Antarctica. Geology 22, 23–26 (1994).

[b58] Aspila K. I., Agemian H. & Chau A. S. Y. Semiautomated method for determination of inorganic, organic and total phosphate in sediments. Analyst 101, 187–197 (1976). 1259177 10.1039/an9760100187

[b59] Xiang X. *et al.* Rapid recovery of soil bacterial communities after wildfire in a Chinese boreal forest. Sci. Rep. 4, 3829, doi: 10.1038/srep03829 (2014). 24452061 PMC3899593

[b60] Muyzer G., Dewaal E. C. & Uitterlinden A. G. Profiling of complex microbial populations by denaturing gradient gel electrophoresis analysis of polymerase chain reaction-amplified genes coding for 16S rRNA. Appl. Environ. Microbiol. 59, 695–700 (1993). 7683183 10.1128/aem.59.3.695-700.1993 PMC202176

[b61] Shi Y. *et al.* Vegetation-Associated Impacts on Arctic Tundra Bacterial and Microeukaryotic Communities. Appl. Environ. Microbiol. 81, 492–501 (2015). 25362064 10.1128/AEM.03229-14 PMC4277566

[b62] Caporaso J. G. *et al.* QIIME allows analysis of high-throughput community sequencing data. Nat Meth 7, 335–336 (2010). 10.1038/nmeth.f.303 PMC3156573 20383131

[b63] Reeder J. & Knight R. Rapidly denoising pyrosequencing amplicon reads by exploiting rank-abundance distributions. Nat. Meth 7, 668–669 (2010). 10.1038/nmeth0910-668b PMC2945879 20805793

[b64] Edgar R. C. Search and clustering orders of magnitude faster than BLAST. Bioinformatics 26, 2460–2461 (2010). 20709691 10.1093/bioinformatics/btq461

[b65] DeSantis T. Z. *et al.* NAST: a multiple sequence alignment server for comparative analysis of 16S rRNA genes. Nucleic Acids Res. 34, W394–W399 (2006). 16845035 10.1093/nar/gkl244 PMC1538769

[b66] Caporaso J. G. *et al.* PyNAST: a flexible tool for aligning sequences to a template alignment. Bioinformatics 26, 266–267 (2010). 19914921 10.1093/bioinformatics/btp636 PMC2804299

[b67] Wang Q., Garrity G. M., Tiedje J. M. & Cole J. R. Naive Bayesian classifier for rapid assignment of rRNA sequences into the new bacterial taxonomy. Appl. Environ. Microbiol. 73, 5261–5267 (2007). 17586664 10.1128/AEM.00062-07 PMC1950982

[b68] Faith D. P. Conservation evaluation and phylogenetic diversity. Biol. Conserv. 61, 1–10 (1992).

[b69] Eisen M. B., Spellman P. T., Brown P. O. & Botstein D. Cluster analysis and display of genome-wide expression patterns. Proc. Natl Acad. Sci. USA 95, 14863–14868 (1998). 9843981 10.1073/pnas.95.25.14863 PMC24541

[b70] De’Ath G. Multivariate regression trees: a new technique for modeling species-environment relationships. Ecology 83, 1105–1117 (2002).

[b71] Xie Z. Q. In Lake sediment and change of ecological environment of penguin on Ardley Island in Antarctica [ Xie Z. Q. (ed.)][75–81](The dissertation for doctor’s degree from University of Science and Technology of China, 2001).

