# Supplementary material for: Bacterial diversity is strongly associated with historical penguin activity in an Antarctic lake sediment profile
Source: Sci Rep. 2015 Nov 25;5:17231. doi: 10.1038/srep17231 (PMC4658551; doi:10.1038/srep17231)
Supplement: Supplementary Information [file srep17231-s1.doc]

**Supplementary Material**

**Bacterial diversity is strongly associated with historical penguin activity in an Antarctic lake sediment profile**

Renbin Zhu^1^,*, Yu Shi^2^, Dawei Ma^1^, Can Wang^1^, Hua Xu^2^, and Haiyan Chu^2^*

^1^Institute of Polar Environment, School of Earth and Space Sciences, University of Science and Technology of China, Hefei city, Anhui Province 230026, PR China

^2^State Key Laboratory of Soil and Sustainable Agriculture, Institute of Soil Science, Chinese Academy of Sciences, Nanjing, Jiangsu Province 210008, PR China

***Corresponding authors:** Renbin Zhu, Tel.: 86-551-63606010; E-mail: zhurb@ustc.edu.cn; Haiyan Chu, Tel.: 86-25-86881356; E-mail: [hychu@issas.ac.cn](mailto:hychu@issas.ac.cn)

**Table S1** Correlations between the biogeochemical properties in the sediment from Lake Y2, in maritime Antarctica.

| Biogeochemical properties | TC | TN | TP | pH | Cu | Zn | Sr | Ba | Fe | Mn | Mg | Al |
| --- | --- | --- | --- | --- | --- | --- | --- | --- | --- | --- | --- | --- |
| TC | 1 | 0.964^**^ | 0.945^**^ | 0.512^**^ | 0.982^**^ | 0.984^**^ | 0.957^**^ | 0.855^**^ | -0.961^**^ | -0.919^**^ | -0.971^**^ | -0.932^**^ |
| TN |  | 1 | 0.850^**^ | 0.315 | 0.950^**^ | 0.953^**^ | 0.886^**^ | 0.727^**^ | -0.873^**^ | -0.808^**^ | -0.890^**^ | -0.940^**^ |
| TP |  |  | 1 | 0.635^**^ | 0.936^**^ | 0.938^**^ | 0.945^**^ | 0.897^**^ | -0.968^**^ | -0.950^**^ | -0.971^**^ | -0.873^**^ |
| pH |  |  |  | 1 | 0.476^**^ | 0.476^**^ | 0.581^**^ | 0.657^**^ | -0.654^**^ | -0.677^**^ | -0.632^**^ | -0.378^*^ |
| Cu |  |  |  |  | 1 | 0.996^**^ | 0.966^**^ | 0.829^**^ | -0.958^**^ | -0.914^**^ | -0.968^**^ | -0.950^**^ |
| Zn |  |  |  |  |  | 1 | 0.961^**^ | 0.845^**^ | -0.962^**^ | -0.918^**^ | -0.972^**^ | -0.952^**^ |
| Sr |  |  |  |  |  |  | 1 | 0.855^**^ | -0.970^**^ | -0.940^**^ | -0.965^**^ | -0.879^**^ |
| Ba |  |  |  |  |  |  |  | 1 | -0.904^**^ | -0.911^**^ | -0.903^**^ | -0.737^**^ |
| Fe |  |  |  |  |  |  |  |  | 1 | 0.979^**^ | 0.993^**^ | 0.897^**^ |
| Mn |  |  |  |  |  |  |  |  |  | 1 | 0.974^**^ | 0.854^**^ |
| Mg |  |  |  |  |  |  |  |  |  |  | 1 | 0.918^**^ |
| Al |  |  |  |  |  |  |  |  |  |  |  | 1 |

Note: **Correlation is significant at the 0.01 level (2-tailed). *Correlation is significant at the 0.05 level (2-tailed).

**Table S2 The number of 454 sequences found the subsamples of the sediment core collected from Lake Y2, in maritime Antarctica.**

| Sediment depths (cm) | Sample No. | Sequence |
| --- | --- | --- |
| 2 | PC2 | 2935 |
| 3 | PC3 | 7096 |
| 4 | PC4 | 6951 |
| 5 | PC5 | 6416 |
| 6 | PC6 | 7442 |
| 7 | PC7 | 6311 |
| 8 | PC8 | 4778 |
| 9 | PC9 | 8351 |
| 10 | PC10 | 2425 |
| 12 | PC12 | 8315 |
| 14 | PC14 | 7878 |
| 16 | PC16 | 8395 |
| 18 | PC18 | 3928 |
| 20 | PC20 | 7774 |
| 22 | PC22 | 7337 |
| 24 | PC24 | 8039 |
| 26 | PC26 | 8161 |
| 28 | PC28 | 6809 |
| 30 | PC30 | 12189 |
| 32 | PC32 | 10173 |
| 34 | PC34 | 10052 |
| 36 | PC36 | 9104 |
| 38 | PC38 | 9916 |
| 40 | PC40 | 6992 |
| 42 | PC42 | 5564 |
| 44 | PC44 | 9354 |
| 46 | PC46 | 8387 |

**Figure S1** Changes in the relative abundance of the dominant phyla, or classes (of Proteobacteria), with depth in the sediments of Lake Y2.

**Figure S2** The relative abundances of the dominant phyla down the sediment profile in Lake Y2. Note: the sediment core (PC) was divided into 45 subsamples at 1.0 cm intervals for the upper 45 cm, and then one subsample for the bottom 46–50 cm. Relative abundances of the dominant phyla in this figure are shown within each 1.0 cm layer in the top 10 cm of the sediment, and then for every other subsample (i.e. at 2.0 cm intervals), for the layers from 10–46 cm depth.

**Table S3** Correlations among the dominant bacterial phyla in the sediment from Lake Y2, in maritime Antarctica.

| Bacterial phyla | Actinobacteria | Gemmatimonadetes | Bacteroidetes | Gammaproteobacteria | Acidobacteria | Chloroflexi | Alphaproteobacteria | Deltaproteobacteria | Betaproteobacteria |
| --- | --- | --- | --- | --- | --- | --- | --- | --- | --- |
| Actinobacteria | 1 | 0.349 | 0.025 | 0.055 | 0.233 | 0.113 | 0.223 | -0.210 | -0.481^**^ |
| Gemmatimonadetes |  | 1 | 0.117 | 0.249 | 0.558^**^ | 0.249 | 0.470^*^ | -0.386^*^ | -0.740^**^ |
| Bacteroidetes |  |  | 1 | 0.474^*^ | 0.213 | 0.352 | 0.180 | -0.575^**^ | -0.475^*^ |
| Gammaproteobacteria |  |  |  | 1 | 0.255 | 0.180 | 0.526^**^ | -0.554^**^ | -0.531^**^ |
| Acidobacteria |  |  |  |  | 1 | 0.663^**^ | 0.494^**^ | -0.453^*^ | -0.763^**^ |
| Chloroflexi |  |  |  |  |  | 1 | 0.450^*^ | -0.347 | -0.584^**^ |
| Alphaproteobacteria |  |  |  |  |  |  | 1 | -0.415^*^ | -0.736^**^ |
| Deltaproteobacteria |  |  |  |  |  |  |  | 1 | 0.588^**^ |
| Betaproteobacteria |  |  |  |  |  |  |  |  | 1 |

**Figure S3** The relationship between the bacterial OTU richness and total carbon, total nitrogen and typical elements of penguin guano, in Lake Y2.

**Figure S4** The relationship between sediment bacterial phylogenetic diveristy and total carbon, total nitrogen and typical elements of penguin guano, in Lake Y2.

**Figure S5** The relationship between sediment bacterial OTU richness and phylogenetic diversity, and Al, Fe, Mn and Mg in Lake Y2.

**Figure S6** The location of the study area and sampling site of the lake sediment core: (a) the red dot indicates location of the study area in maritime Antarctica; and (b) the location of the sediment core (PC) sampling site, from Lake Y2 on Ardley Island. *The map was drawn using Microsoft Excel 2010 and then converted to eps format using Microsoft Office Visio 2007.

**a**

**Fildes Peninsula**

**Ardley Island**

**Y2**

**b**
